# Development of dental caries and risk factors between 1 and 7 years of age in areas of high risk for dental caries in Stockholm, Sweden

**DOI:** 10.1007/s40368-021-00642-1

**Published:** 2021-06-09

**Authors:** M. Anderson, G. Dahllöf, A. Warnqvist, M. Grindefjord

**Affiliations:** 1 Division of Orthodontics and Pediatric Dentistry, Department of Dental Medicine, Karolinska Institutet, Stockholm, Sweden; 2 Department of Pediatric Dentistry, Folktandvården Eastmaninstitutet, Folktandvården Stockholms Län AB, Stockholm, Sweden; 3 Center of Pediatric Oral Health, Stockholm, Sweden; 4 Division of Biostatistics, Institute of Environmental Medicine, Karolinska Institutet, Stockholm, Sweden; 5 Pedodonti, Folktandvården Eastmaninstitutet, Dalagatan 11, 10231 Stockholm, Sweden; 6 Center for Oral Health Services and Research, TkMidt, Trondheim, Norway

**Keywords:** Early childhood caries, ICDAS, Preschool children, Prevention, Topical fluoride, Progression

## Abstract

**Purpose:**

To explore caries predictors at age 1 year and caries development at ages 5 and 7 years in two groups of children following different fluoride-based preventive programs.

**Methods:**

We conducted a prospective cluster-randomized controlled intervention trial with two parallel arms comparing two prevention programs: one program included fluoride varnish applications every 6 months, the other did not; otherwise, the programs were the same. Participants were 1- and 3-year-old children enrolled at 23 dental clinics in high-risk areas in Stockholm, Sweden. The baseline examination included structured interviews. Caries data were extracted from dental records. The primary outcome measures were ICDAS 1–6 > 0 at baseline (age 1 year) and defs > 0 at ages 2, 3, 5, and 7 years. The secondary outcome measure at age 7 was DFS > 0.

**Results:**

Continuous caries development occurred: defs > 0 in 23% at 5 years and in 42% at 7 years. We found no difference in caries development between children who had or had not received fluoride varnish as toddlers. At age 1-year, significant predictors for dental caries in later preschool years were immigrant background, family income, and sweets consumption. Fluoride toothpaste > once a day at 1 year had an OR < 1 for defs > 0 at 5- and 7 years.

**Conclusions:**

For toddlers, fluoride varnish does not seem to be an adequate prevention tool. Brushing with fluoride toothpaste from 1 year of age could not arrest caries development. Immigrant background was the strongest predictor. A new toolbox as well as collaborative upstream actions for reducing free-sugar intake are needed.

## Introduction

Dental caries continues to be a major oral health problem. According to the 2017 *Global Burden of Disease Study*, 532 million children suffer from dental caries in their deciduous teeth (GBD 2018). The prevalence of caries in preschool children varies (Kassebaum et al. 2015). Reports have observed numbers between 0.5% and 75% in developed countries (Anderson et al. 2016; Douglass et al. 2001; Koch et al. 2017; Phipps et al. 2012; Stecksén-Blicks et al. 2014). Children within a country may be unevenly affected (Schwendicke et al. 2015). Socio-economic determinants and individual daily habits play integral roles (Watanabe et al. 2014). Studies have identified immigrant background as well as maternal education level to be caries predictors in preschool children (Grindefjord et al. 1995b; Grindefjord et al. 1996; Skeie et al. 2008; Wendt et al. 1996). Karjalainen and co-workers found in a recent published study that a high sugar intake at 3 years of age was significantly associated with a high risk of caries as well as high mutans streptococci counts (Karjalainen et al. 2015). Overall past caries experience, however, has been shown to be the best predictor for continued caries development and the best predictor overall (Burt and Pai 2001; Mejàre et al. 2014).

In Sweden, dental healthcare is free of charge for children, and the first examination with a dentist generally occurs at 3 years of age (Dental Service in Stockholm Ltd 2018). Previously, we conducted a 2-year intervention trial in toddlers. The program began at 1 year of age, with the aim of stopping disease development at an early stage. The cluster-randomized trial compared two caries preventive programs: one with fluoride varnish applications and one without. The trial was conducted in high-risk areas (areas with high prevalence of dental caries in children) in Stockholm. When the children were 3 years old, we found no significant differences in caries prevalence between the two programs (Anderson et al. 2016).

The aim of the present trial was to study caries development at ages 5 and 7 years as well as predictors in the children at 1 year of age who had followed the two different preventive programs from 1 to 3 years of age. Our first hypothesis was that caries development is the same at ages 5 and 7 years, independent of the preventive program the children had followed as toddlers. Our second hypothesis was that predictors for caries development change during preschool years.

## Materials and methods

### Settings and participants

The Stop Caries Stockholm (SCS) study was conducted in 23 dental clinics located in areas with a multicultural population and families predominately of medium or low socioeconomic status. All children born in these areas between 1 January and 31 December 2010 in Stockholm County were invited to participate. The intervention part lasted for years, between March 2011 and March 2014.

**Fig. 1:**
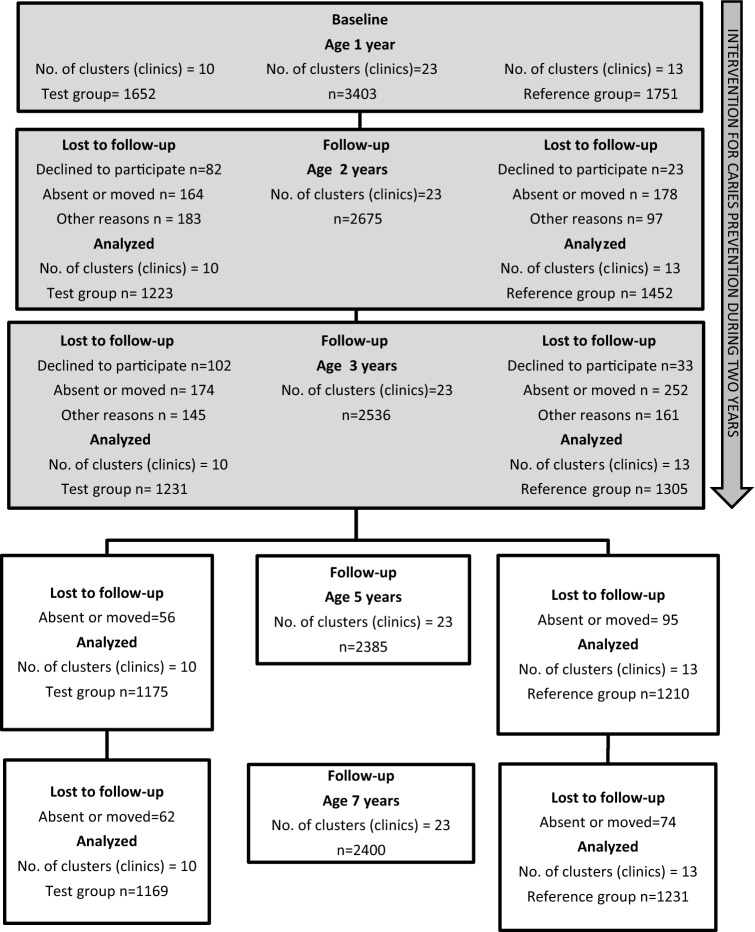
Caries development (defs > 0) from 1 to 7 years of age (%)

The study was cluster-randomized and had a parallel design. Interventions occurred when the children where between 1 and 3 years of age. A first analysis was made following the intervention when the children were 3 years old. After this, the children received the standard national intervention. In the present study, we analyzed data from the same cohort at ages 1 year (baseline), and 2, 3, 5, and 7 years. At 5 and 7 years, all children in the previous study (Anderson et al. 2016) who had been examined at 3 years of age were invited to join the present trial; because data were collected from the dental records, children who had moved could be included. At 5 years, 70% (*n* = 2385) and at 7 years, 71% (*n* = 2400) of the children who had completed the examination at 3 years of age were examined. The flowchart in Fig. 1 shows how many dropped out and why over the course of the 6 years. The Regional Ethics Committee in Stockholm approved the study (Daybook no. [Dnr] 2010/1956 and Daybook no. [Dnr] 2016/1240-32, www. controlled-trials.com [ISRCTN35086887]).

### Caries prevention

The SCS study included a reference group and a test group. Between ages 1 and 3 years, all children in the study received a standard caries prevention program. This intervention was given once a year to the reference group and twice a year to the test group. Additionally, the test group received fluoride varnish treatments (Duraphat^®^, 22.6 mg of fluoride per ml, Colgate-Palmolive) at all intervention sessions. For more details, see Anderson et al. (2016). Between 3 and 7 years of age, the children were enrolled in the same caries preventive program. All dental caregivers for children must deliver the caries prevention program that the Stockholm County Council introduced in 2004 (2018).

### Questionnaire

At the 1-, 2-, and 3-year annual examinations in the present study, the examiner interviewed the parent, completing a survey questionnaire. An interpreter was available to assist parents who had difficulties with the Swedish language (Anderson 2017).

### Outcome measures

Data collected during examinations in the SCS trial were used to determine development of dental caries using the International Caries Detection and Assessment System (ICDAS, a clinical scoring system, range 1–6) or defs (decayed, extracted, filled surfaces in primary teeth) when the children were 1, 2, and 3 years old (Anderson 2017). In the SCS trial, the ICDAS system (Ismail et al. 2007) was used to score dental caries. ICDAS dental caries scores were translated to the WHO criteria for cavitation as recommended by Braga and co-workers (Braga et al. 2009). A tooth surface with an ICDAS score of 3−6 was considered decayed. The examiners also recorded whether a tooth had been filled or was missing because it had failed to erupt, had been extracted due to dental caries, or was missing for other reasons. This has been described in detail elsewhere (Anderson et al. 2016). Dental caries (defs/decayed, filled surfaces in permanent teeth [DFS]) data for each child at ages 5 and 7 years were retrieved from the digitalised patient records of public and private dental clinics serving the catchment areas. Only data from children in the SCS trial who had been examined at the age of 3 years were collected, the children who did not attend this examination were considered as drop-outs. In Sweden, clinicians determine clinical dental status on the tooth level according to WHO recommendations. From 5 years of age, bitewing radiographs are recommended and proximal lesions clearly extending into the dentine are registered as decayed (Folktandvården Stockholms län AB 2004; Koch 1967; Swedish National Board of Health and Welfare 2010a).

The primary outcome measure at baseline (1-year-olds) was presence of caries, ICDAS 1–6 (i.e., an ICDAS score > 0). For the other ages, the primary outcome measure was defs > 0 (ICDAS 3–6). At 7 years of age, the secondary outcome was caries defined as DFS > 0.

Before examining the children when they were 7 years old, the examiners (a team comprising a dentist and a dental hygienist) completed an e-learning session concerning caries registration. This training consisted of a Power Point^®^ presentation of information on optimal conditions for caries registration and calibration exercises for diagnosing dental caries.

### Statistical analyses

All data were processed in Microsoft Excel 2016, in the Statistical Package for the Social Sciences (SPSS, IBM Software Statistics, version 26.0) and in Stata version 15 (StataCorp, release 15.0). Table 1 presents the dichotomized, continuous and categorical variables that were used in the statistical models.

**Table 1:** Baseline characteristics at age 1 year (*n* = 3403)

Variables	Value	%
Gender		
Female Male	0 1	51.7 48.3
Immigrant background		
No Yes	0 1	21.5 78.5
Family income		
≤ SEK20,000/month > SEK20,000/month	0 1	39.5 60.5
Maternal education		
≤ 9 years > 9 years	0 1	21.4 78.6
Tooth brushing (fluoride toothpaste)		
< 1/day ≥ 1/day	0 1	44.9 55.1
Tooth brushing (fluoride toothpaste)		
< 2/day ≥ 2/day	0 1	66.8 33.2
Consumption of sugar-containing beverages		
Never ≥ 1/day	0 1	37.1 62.9
Sweets consumption		
Never ≥ 1/week	0 1	70.4 29.6
Gingivitis		
No Yes	0 1	80.0 20.0
ICDAS 1–6		
No Yes	0 1	94.8 05.2
defs > 0		
No Yes	0 1	99.4 00.6

*ICDAS* International Caries Detection and Assessment System, *defs* decayed, extracted, filled surfaces in primary teeth

Differences in proportion of caries between the two exposure groups was determined with the χ^2^ test. Each year was tested separately. A univariate logistic regression model was used to determine the extent to which the independent variables, observed at 1 year of age, were able to differentiate between the presence and absence of caries at 1, 2, 3, 5, and 7 years of age. A multivariate logistic regression model was used to estimate the impact of a risk factor when controlling for other risk factors.

Cumulative risk was estimated in logistic regression analyses using the accumulation of risk factors as categories. The category “zero risk factors” was used as a reference when estimating the probabilities of caries for various risk combinations. A clustered robust covariance matrix estimate was used for all logistic regression models to control for within-cluster correlations on the clinic level. The level of statistical significance was set at 0.05.

Sensitivity and specificity as well as positive and negative predictive values were determined according to the definitions commonly used in the literature (Trevethan 2017).

### Missing data

To investigate if missingness could be assumed to be at random, the baseline values for different study population characteristics were compared between those who completed the study and those who dropped out before 7 years of age. Differences were tested with a χ^2^ test. The proportions of risk factors listed in Table 2 were tested. No significant differences were found. The outcome measures were also tested regarding missing data, and no significant differences were found except for dental caries (defs) at 7 years of age. Of the children with dental caries at 3 years of age, 7% (*n* = 27) did not participate in the 7-year examination. The number of missing children with defs > 0, however, were evenly distributed between the test and the reference group (n.s.).

**Table 2:** Caries-free children at 5 (defs = 0) and 7 (defs = 0, DFS = 0) years in the test and reference groups

	Total	Study group		*p* value
		Test	Reference	
Age 5 years				
Children (*n*)		1175	1210	
defs = 0 (%, *n*)	76.7 (1830)	78.0 (917)	75.5 (913)	0.99
Age 7 years				
Children (*n*)		1169	1231	
defs = 0 (%, *n*)	58.0 (1392)	59.5 (695)	56.6 (697)	0.90
DFS = 0 (%, *n*)	92.3 (2181)	92.1 (1076)	92.4 (1105)	0.99

*defs* decayed, extracted, filled surfaces in primary teeth, *DFS* decayed, filled surfaces in permanent teeth

## Results

### Caries development

**Fig. 2:**
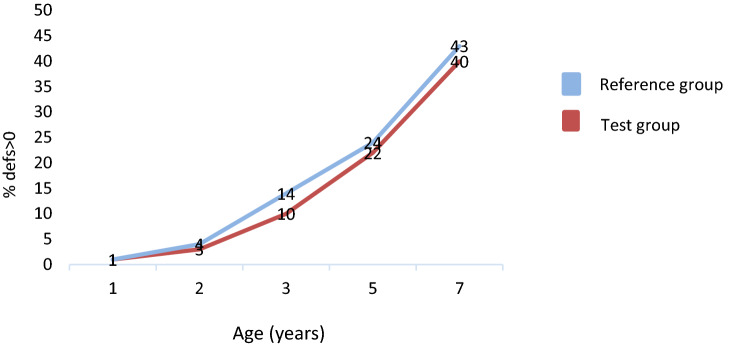
Flowchart of randomized children and reasons for dropping out at ages 2, 3, 5, and 7 years. Intervention times are also presented

Figure 2 presents caries development from 1 to 7 years of age. The proportion of children with caries follows an increasing trend in both the test and reference groups. Dental caries defs > 0 (ICDAS 3–6) was found in 1% of the children at age 1 year and 4% at age 2, 12% at age 3, 23% at age 5, and 42% at age 7 years. Only 22 of the 1-year-olds in the trial had defs > 0 (ICDAS 3–6) and 177, an ICDAS of 1–6. Between ages 5 and 7 years, the proportion of children with caries extending into the dentine nearly doubled. At 7 years of age, 8% had developed caries (DFS > 0) in their permanent teeth. Caries were equally distributed between boys and girls.

### Frequency of tooth brushing

Fifty-five per cent of the 1-year-olds brushed their teeth daily with fluoride toothpaste, as did 87% of the 2-year-olds and 92% of the 3-year-olds.

### Sugar intake at different ages

Parents reported consumption of sweets for 30% of the 1-year-olds, 75% of the 2-year-olds, and 90% of the 3-year-olds. Sugar-containing beverages were consumed by 63% of the 1-year-olds, 85% of the 2-year-olds, and 87% of the 3-year-olds.

### Risk factors for caries development

Comparisons of baseline characteristics of 1-year-olds with and without dental caries (defs > 0; Table 1) in a univariate analysis showed that several socioeconomic status (SES) factors such as immigrant background, maternal education ≤ 9 years, and family income of ≤ SEK20,000 a month were significantly associated with the development of dental caries. Consumption of sugar-containing beverages as well as sweets consumption ≥ 1/week and gingivitis were also significantly associated with caries development. Use of fluoride toothpaste at least once a day at 1 year of age was significantly associated with a lower development of dental caries at 1, 2, 3, 5, and 7 years of age (Table 3). The risk of future caries development was lower for children who used fluoride toothpaste twice a day.

**Table 3:** Univariate logistic regression analyses of baseline variables at 1 year of age and children with (yes) and without (no) dental caries at ages 1, 2, 3, 5, and 7 years. Presence of dental caries was defined as an ICDAS of 1–6 at 1 year of age and a defs > 0 at ages 2, 3, 5, and 7 years

Risk factors	Presence of dental caries				
	(Age)				
	1 year	2 years	3 years	5 years	7 years
(Yes/no; *n*)	(177/3226)	(172/2503)	(573/2536)	(555/1830)	(1008/1392)
Immigrant background					
Yes/no (%)	94/78	94/77	93/77	91/75	87/73
OR	3.63***	4.83***	4.56***	3.43***	2.42***
95% CI	1.88–6.98	2.46–9.50	2.74–7.58	2.39–4.93	1.93–3.07
Family income (< SEK20,000/month)					
Yes/no (%)	58/39	61/38	56/38	54/35	48/33
OR	2.24***	2.48***	2.14***	2.19***	1.85***
95% CI	1.44–3.49	1.72–3.57	1.68–2.72	1.70–2.83	1.50–2.28
Maternal education (≤ 9 years)					
Yes/no (%)	31/21	32/21	33/21	32/19	27/19
OR	1.73***	1.77**	1.84***	2.10***	1.54***
95% CI	1.06–2.80	1.17–2.68	1.41–2.40	1.67–2.66	1.19–2.00
Tooth brushing (≥ 1/day)					
Yes/no (%)	54/56	48/56	45/57	47/58	50/59
OR	0.95	0.73	0.63***	0.65***	0.67***
95% CI	0.69–1.35	0.52–1.03	0.46–0.83	0.55–0.72	0.58–0.79
Tooth brushing (≥ 2/day)					
Yes/no (%)	30/33	22/34	23/35	24/36	28/37
OR	0.85	0.55**	0.56***	0.55***	0.68***
95% CI	0.58–1.26	0.36–0.84	0.41–0.76	0.47–0.62	0.57–0.80
Sweets (≥ 1/week)					
Yes/no (%)	41/29	40/29	44/28	42/26	38/24
OR	1.72***	1.65**	2.06***	1.98***	1.88***
95% CI	1.32–2.28	1.14–2.38	1.63–2.60	1.54–2.55	1.46–2.41
Sugar-containing beverages (yes)					
Yes/no (%)	68/63	70/62	77/62	70/62	68/61
OR	1.28**	1.44**	2.01***	1.36**	1.38**
95% CI	1.02–1.60	1.05–1.99	1.60–2.55	1.08–1.80	1.09–1.74
Gingivitis (yes)					
Yes/no (%)	23/4	13/4	9/4	7/4	5/5
OR	7.74***	3.32***	2.43**	1.59*	1.14
95% CI	3.96–15.11	1.72–6.39	1.47–4.02	1.00–2.43	0.71–1.82
ICDAS 1–6 (at 1 year of age)					
Yes/no (%)	–	4/36	4/19	4/11	4/9
OR	–	12.9***	5.85	2.84***	2.23***
95% CI	–	7.98–20.85	0.08–9.73	1.84–4.38	1.57–3.18

*OR* crude odds ratio, *CI* confidence interval

*p < 0.05, **p < 0.01, ***p < 0.001

### Predictors at 1 year of age during the preschool years

Variables that were significantly associated with caries development at 1 year of age were analyzed in a multivariate logistic regression analysis. When tooth brushing was the independent variable, only the variable with the highest odds ratio (OR) was included in the multivariate analysis as the two factors were strongly associated with each other. The independent impact of predictors for the development of dental caries (ICDAS 1–6) at 1 year of age and the development of dental caries (defs > 0) at succeeding time points was evaluated at ages 2, 3, 5, and 7 years (Table 4). Immigrant background was a significant predictor at all time points but OR was highest at 1 year of age and lowest at 7 years. The OR of the other two significant SES predictors—family income < SEK20,000 and maternal education < 9 years—also dropped in succeeding examinations, but not to the same extent as for immigrant background. The OR for the significant predictors, sweets consumption and consumption of sugar-containing beverages at 1 year of age was higher at later time points. The OR for tooth brushing ≥ 1/day, on the other hand, decreased at later time points, including the older preschool years (Table 4).

**Table 4:** Multivariate logistic regression analyses with presence of dental caries (defined as an ICDAS of 1–6 at 1 year of age and a defs > 0 at 2, 3, 5, and 7 years) as the dependent variable and various risk factors as the independent variables

Risk factors	Presence of dental caries				
	Age				
	1 year	2 years	3 years	5 years	7 years
Immigrant background					
OR	3.20***	2.62**	2.84***	2.25***	1.71***
95% CI	1.61–6.38	1.27–5.42	1.57–5.14	1.55–3.27	1.34–2.69
Family income (< 20,000 SEK/month)					
OR	1.80**	1.60**	1.41**	1.56**	1.46***
95% CI	1.24–2.07	1.21–2.30	1.12–1.78	1.17–2.10	1.22–1.74
Maternal education (≤ 9 years)					
OR	1.35	1.25	1.29**	1.50***	1.14
95% CI	0.84–2.18	0.83–1.88	1.01–1.66	1.21–1.86	0.89–1.48
Tooth brushing (≥ 1/day)					
OR	1.37	1.01	0.74	0.80**	0.78**
95% CI	0.90–2.07	0.71–1.42	0.53–1.03	0.69–0.92	0.67–0.91
Sweets (≥ 1/week)					
OR	1.51*	1.36	1.58***	1.69***	1.69***
95% CI	1.03–2.20	0.88–2.12	1.24–1.99	1.34–2.13	1.30–2.13
Sugar-containing beverages (yes)					
OR	0.96	1.36	1.63***	1.12	1.12
95% CI	0.71–1.29	0.88–1.41	1.24–2.14	0.87–1.43	0.89–1.40
Gingivitis (yes)					
OR	8.23***	1.31	1.25	1.11	0.87
95% CI	4.09–16.60	0.62–2.78	0.74–2.13	0.67–1.83	0.53–1.43
ICDAS 1–6 (at 1 year of age)					
OR	–	10.05***	4.82***	2.25***	1.90***
95% CI	–	6.16–16.38	3.01–7.73	1.54–3.29	1.34–2.69

*ICDAS* International Caries Detection and Assessment System, *defs* decayed, extracted, filled surfaces in primary teeth, *OR* odds ratio, *CI* confidence interval

*p < 0.05, **p < 0.01, ***p < 0.001

Table 5 presents the sensitivity, specificity, and positive and negative predictive values for the SES predictors at 1 year of age. We can see that the predictive values for caries development changes for each predictor as the children grow older while caries prevalence increases. Immigrant background had the highest sensitivity (93.8–86.8%); specificity increased over time but was still low (44.8%) at age 7. Maternal education ≤ 9 years was the predictor with the highest specificity (78.8–81.1%), but sensitivity was low and never above 32.9% calculated at 5 year of age. Sweets consumption (≥ 1/week) at 1 year of age could identify 69.9% of the children who had developed dental caries at 5 years of age and 52.8% at 7 years. Negative predictive values, however, were higher overall for sweets consumption.

**Table 5:** Predictive values (%) for caries-associated variables at 2, 3, 5, and 7 years of age

Risk factors	Age				
	1 year	2 years	3 years	5 years	7 years
Immigrant background					
SE	92.7	94.2	93.8	91.2	86.8
SP	22.3	22.9	23.1	24.9	44.8
PV+	06.2	05.4	14.4	26.9	39.3
PV−	98.2	98.8	96.4	90.3	63.0
Family income (< SEK20,000/month)					
SE	58.4	60.5	56.0	54.3	40.8
SP	61.5	61.8	62.7	64.8	66.7
PV+	07.8	06.9	17.3	31.6	50.7
PV−	96.4	97.1	91.1	82.6	64.2
Maternal education (≤ 9 years)					
SE	31.3	32.2	32.7	32.9	26.6
SP	79.2	78.8	79.1	81.1	81.0
PV+	07.7	06.7	17.7	34.6	50.2
PV−	95.4	96.1	89.5	79.9	60.5
Tooth brushing					
SE	45.8	77.7	55.1	52.9	50.3
SP	55.5	34.2	56.5	58.0	59.4
PV+	07.3	05.2	14.9	27.7	47.3
PV –	94.9	97.0	90.1	80.2	62.3
Tooth brushing (≥ 2/day)					
SE	70.1	52.1	77.0	75.9	71.6
SP	33.4	55.7	34.8	36.5	37.0
PV+	05.5	05.2	14.0	26.7	45.2
PV−	95.3	96.1	90.1	83.3	64.3
Sweets (≥ 1/week)					
SE	41.2	39.7	44.3	41.6	37.6
SP	71.0	71.4	72.0	73.6	75.7
PV+	07.3	06.1	18.0	69.6	52.8
PV−	95.6	96.2	90.3	80.6	62.6
Sugar-containing beverages (yes)					
SE	68.2	70.2	76.5	69.6	68.1
SP	37.4	38.0	38.1	37.8	39.3
PV+	05.7	05.0	14.6	25.4	44.8
PV−	95.5	96.5	92.1	80.4	63.0
Gingivitis (yes)					
SE	23.3	13.3	09.3	06.8	05.2
SP	96.2	95.6	95.9	95.6	95.5
PV+	25.6	12.4	23.9	32.2	45.1
PV−	95.7	95.9	88.5	77.1	58.1
ICDAS 1–6 (at 1 year of age)					
SE	–	35.5	19.2	11.4	07.8
SP	–	95.9	96.1	95.7	96.3
PV+	–	28.7	40.4	44.4	60.8
PV−	–	97.0	89.6	78.1	59.0

Dental caries at 1 year of age: ICDAS 1–6. Dental caries at 2, 3, 5, and 7 years: defs > 0

*SE* sensitivity, *SP* specificity, *PV +* positive predictive value, *PV−* negative predictive value, *ICDAS* International Caries Detection and Assessment System, *defs* decayed, extracted, filled

### Cumulative risk

**Fig. 3:**
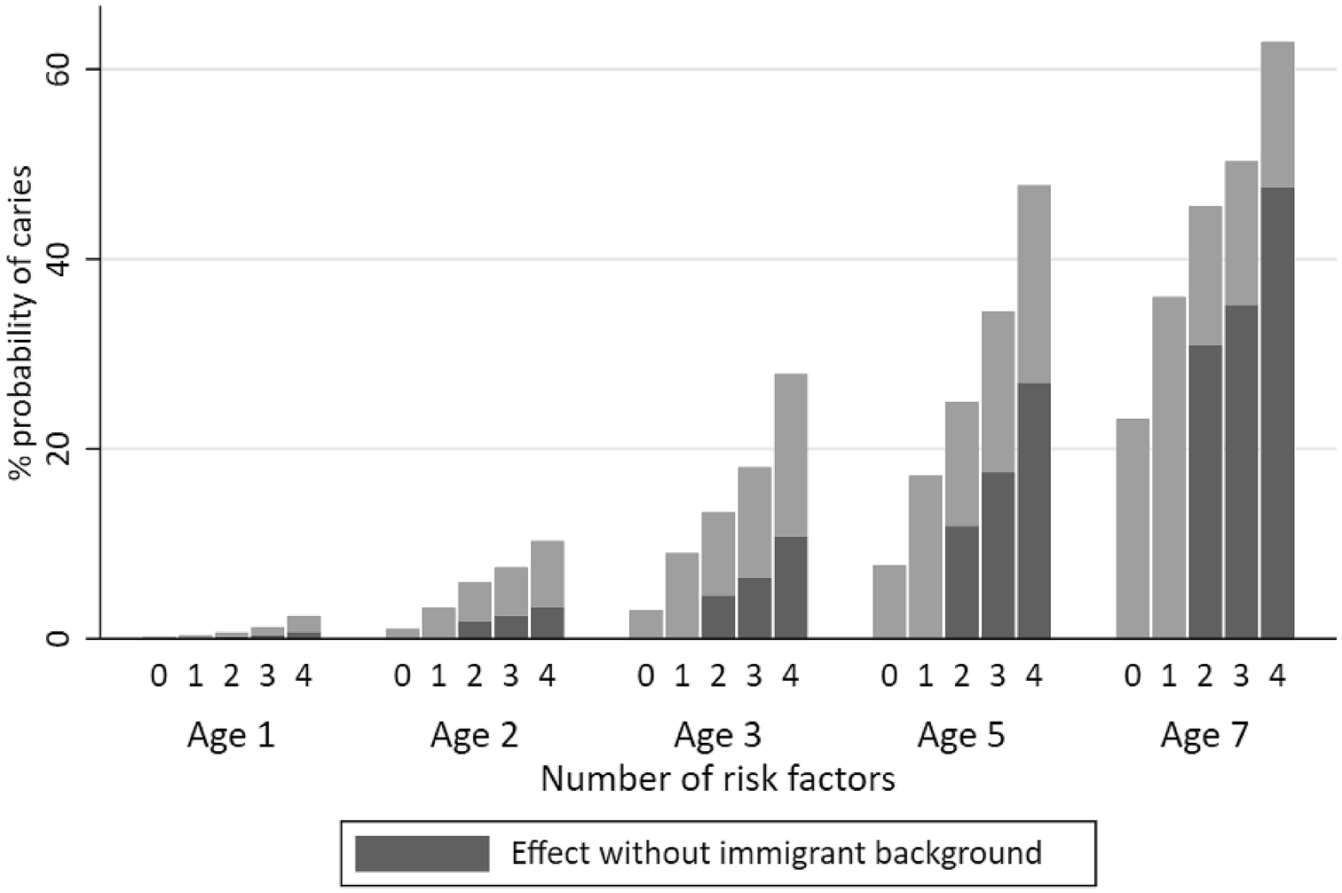
Cumulative probability of defs > 0 at ages 1, 2, 3, 5, and 7 years when none of the risk factors were identified at 1 year of age and when 1, 2, 3, or 4 risk factors were present. 0 = no risk factor, 1 = immigrant background, 2 = immigrant background + family income < SEK20,000/month; 3 = immigrant background + family income < SEK20,000/month + maternal education < 9 years, 4 = immigrant background + family income < SEK20,000/month + maternal education < 9 years + sweets ≥ 1/week. When none of the predictors identified in this study were present, an odds ratio of 1.0 (no risk) was used

An estimation of probability of developing dental caries was made using different profiles of risk factors at 1 year of age for the development of dental caries (defs > 0) at ages 1, 2, 3, 5, and 7 years (Fig. 3). Probability increased with additional risk factors. The probability of having dental caries (defs > 0) with none of the risk factors increased from 0 at 1 year of age to 23.2% at 7 years of age. The probability of having dental caries (defs > 0) with no risk factor present more than doubled between ages 5 and 7 years (2 years = 1.0%, 3 years = 3.0%, 5 years = 7.7%, and 7 years = 23.2%). With all risk factors present (all SES factors and sweets consumption ≥ 1/week), the relative risk of dental caries would be 62.9% at 7 years of age. When combined with immigrant background, sweets ≥ 1/week increases the probability of caries more than any other risk factor between ages 1 and 7 years (Fig. 3).

## Discussion

In the present study, we found that an extended standard program from the age of 1 year that included a fluoride varnish application every 6 months did not reduce the development of dental caries longitudinally in the primary dentition; we observed continuous caries development (defs) and found no significant difference between the two intervention groups at ages 5 and 7 years. Further, predictors for caries development during the later time points including preschool ages could be identified at 1 year of age and their strength varied during the preschool years.

An earlier study found that 29% of the caries-free 1-year-olds had developed dental caries 1 year later and that of those with dental caries at 1 year of age, 92% had developed new lesions (Grindefjord et al. 1995b). The failing of preventing the children from developing dental caries was also found in another well-controlled clinical intervention trial. Of the caries-free children between 2 and 3 years of age, 37% developed dental caries in the following year (Tickle et al. 2017).

An interesting finding in the present study was that the proportion of children with dental caries (defs) nearly doubled between 5 and 7 years of age. We should consider, though, that this caries is not new but rather a sign of the effect that time itself has on caries progression in a caries-active individual (Shwartz et al. 1984).

Designing our intervention trial, based on earlier knowledge and recommendations at that time (Marinho et al. 2002), our beliefs on the supplemental biannual fluoride varnish applications in children from 1 year of age was that they would be effective. But as expected, taking our 2 years of follow-up results into consideration, the semi-annual applications of fluoride varnish did not reduce caries development in the longer term either. Several recent studies have also shown that adding fluoride varnish to a standard prevention program in toddlers is not effective in preventing the development of dental caries (Agouropoulos et al. 2014; Oliveira et al. 2014; Tickle et al. 2017). When applying fluoride varnish in young children, there is a risk of exposure of high serum levels of fluoride (Lockner et al. 2017). If fluoride varnish twice a year is not enough, how many times should it be applied to be effective? Due to the risk of dental fluorosis, sodium fluoride varnish does not seem to be the right tool for preventing dental caries in toddlers.

In this study, an increasing number of children brushed their teeth with fluoride toothpaste during the intervention. This fact has been considered an explanatory factor to the lacking supplementary preventive effect of fluoride varnish. Overall daily use of fluoride toothpaste has been shown to be the most health economic caries preventive intervention (Swedish Agency for Health Technology Assessment 2002).

In the present study, we were able to identify socioeconomic factors, such immigrant background, family income, and maternal education level, as significant predictors for dental caries development. Several other studies have also identified social determinants to be associated with dental caries (Julihn et al. 2018; Mejàre et al. 2014).

Consumption of sweets and sugar-containing beverages at baseline (age 1 year) was found to be significantly associated with the development of dental caries longitudinally. Both daytime and night time consumption of sugary drinks in early childhood has been shown to be important predictors for dental caries later in the primary dentition (Wigen and Wang 2015). In the univariate analysis, the OR was less than 1 at all measurement points for the risk factor daily tooth brushing with fluoride toothpaste. Early, well-functioning homecare routines are stressed as important health-promoting factors.

In a model-based multivariable logistic regression analysis, we studied predictors at 1 year of age for dental caries development throughout the preschool examinations, up to and including age 7. At most examinations, the studied predictors were significant, but the ORs varied. The OR for immigrant background dropped over time but was still the strongest single risk factor in the study cohort. Accumulating risk factors increased the probability of dental caries. No single caries-associated risk factor had sufficient predictive value on its own. Generally, the risk factors were better in correctly identifying healthy individuals than individuals who were developing dental caries. In a similar study as ours, but conducted in a low-risk area in Sweden, the accuracy of predictors 1 year of age for dental caries at 6 years was limited, but the risk assessment was better if several factors were weighted together (Hultquist et al. 2021).

A strength in our study is its prospective cluster-randomized longitudinal design, which allowed us to follow the sequential effect of our interventions starting with eruption of the first primary teeth when the children were 1 year old through age 7 years, which is unusual. Another strength was that we had information on the social determinants from baseline.

Despite supplemental fluorides at an early age, and although 90% of the 3-year-olds brushed their teeth with fluoride toothpaste (Anderson et al. 2016) and parents had early knowledge of risk factors and preventive information, 42% of the children had developed caries in their primary dentition by 7 year of age. Our caries preventive toolbox seems to be outdated and in need of modification.

Bias is always a risk in clinical trials and in ours, non-blinding was one (Schulz and Grimes 2002). Concerning compliance and retention, we found no significant differences in dropout rates between the test and reference groups. A randomization on the individual level is usually preferred. Ours, on the cluster level, was chosen so as not to mix the two interventions, and it also helped us to balance the two groups in the pre-study stage regarding the bias of the intrinsic SES characteristics.

At 5 years of age, 70% of the children in the 3-year examination were examined and at 7 years, 71% of the children. If we consider that the catchment areas had a low SES, and that the majority of the parents had an immigrant background, which is linked to a higher rate of movement (Statistics Sweden 2008; Swedish National Board of Health and Welfare 2010b), retention of the participants in the present study should be considered fair. The drop-outs in our trial did not differ concerning baseline data at 5 or 7 years. Further the number of participating children was high, and the drop-outs were balanced between the test and the reference group.

Instead of looking at risk factors for dental caries, presence of dental caries itself could be considered as a risk factor for identifying the health behavior of the individual. Free sugars above 10% (or even less) of an individual’s total energy intake are precursors to dental caries (Sheiham and James 2015; World Health Organization 2015). We need to work translationally, downstream as well as upstream, to maintain good general health of individuals throughout their lives. Encouraging parents to avoid introducing added sugar before the age of 2 (Pitts et al. 2019) is a good start for both lowering caries prevalence in the population and enhancing the general health of the individual (Vos et al. 2017). Also, to introduce regular tooth brushing with fluoride toothpaste from 1 year of age (Brannemo et al. 2021). Earlier studies on caries progression and ECC prevention emphasize the importance of early intervention, with first dental visit preferably at 1 year of age (Grindefjord et al. 1995a; Wennhall et al. 2008). It is also known that caries preventive programs have the greatest impact in high-risk groups during the period when interventions are ongoing (Brannemo et al. 2021) or most intense (Wennhall et al. 2008). This indicate that more long-term support is needed. Postnatal home visiting programs may be a way to support development and well-being in children and seem to be especially promising in socially high-risk families (Burstrom et al. 2017; Wennhall et al. 2008). But neither dentistry nor primary healthcare can do this alone. More wide-reaching political actions are needed to manage these behavioral- and sugar-consumption issues.

Dental caries is a lifestyle disease. The present study provides evidence that neither applying fluoride varnish in.

## Conclusion

For toddlers, fluoride varnish does not seem to be an adequate prevention tool. Brushing with fluoride toothpaste from 1 year of age could not arrest caries development. Immigrant background was the strongest predictor. A new toolbox as well as collaborative upstream actions for reducing free-sugar intake are needed.
